# Selective Separation of Rare Earth Elements by Nanofiltration Membranes: Mechanisms, Performance, and Perspectives

**DOI:** 10.3390/membranes16080268

**Published:** 2026-08-13

**Authors:** Zhenhua Feng, Wenjie Jiang, Binbin Tang, Xiaojun Yang, Ke Liu, Guangyong Zeng

**Affiliations:** 1 Sichuan Provincial Testing Research Center of Natural Resources (Sichuan Provincial Nuclear Emergency Technical Support Center), Chengdu 610081, China; fengshangzhong86@163.com (Z.F.); jiangwenjie0719@163.com (W.J.); tangbinbin2008@163.com (B.T.); yangxj73_cszx@163.com (X.Y.); 2 College of Materials and Chemistry & Chemical Engineering, Chengdu University of Technology, Chengdu 610059, China; 2023020668@stu.cdut.edu.cn

**Keywords:** rare earth elements, nanofiltration membrane, selective separation, membrane materials, mechanisms and applications

## Abstract

Rare earth elements (REEs) are critical for advanced manufacturing and clean energy, yet their separation remains extremely challenging due to the nearly identical ionic radii of adjacent lanthanides. Conventional solvent extraction, ion exchange, and precipitation methods are limited by their high reagent consumption, slow kinetics, poor selectivity, and environmental burdens. Nanofiltration (NF) offers a green and efficient alternative—operating in the aqueous phase with low energy demand and continuous high throughput. This review systematically summarizes NF-based REE separation. We first elucidate the fundamental mechanisms (size exclusion, Donnan exclusion, dielectric exclusion, and complexation enhancement), and discuss how lanthanide hydration chemistry underpins these synergistic effects. Membrane materials, from commercial to biomimetic, are critically surveyed, with an emphasis on strategies to overcome the trade-off between permeability and selectivity. The impacts of operating conditions and solution chemistry are analyzed, and NF applications ranging from single REE systems to real leachates are assessed. A comparative evaluation positions NF against conventional technologies. Key challenges remain: poor adjacent REE selectivity, membrane fouling, performance loss at high salinity, chemical instability, and a gap between model and real feeds. Future directions include designing high-selectivity membranes, integrating machine learning optimization, establishing standardized protocols, and realizing closed-loop process integration.

## 1. Introduction

### 1.1. Overview of Rare Earth Elements

**Figure 1:**
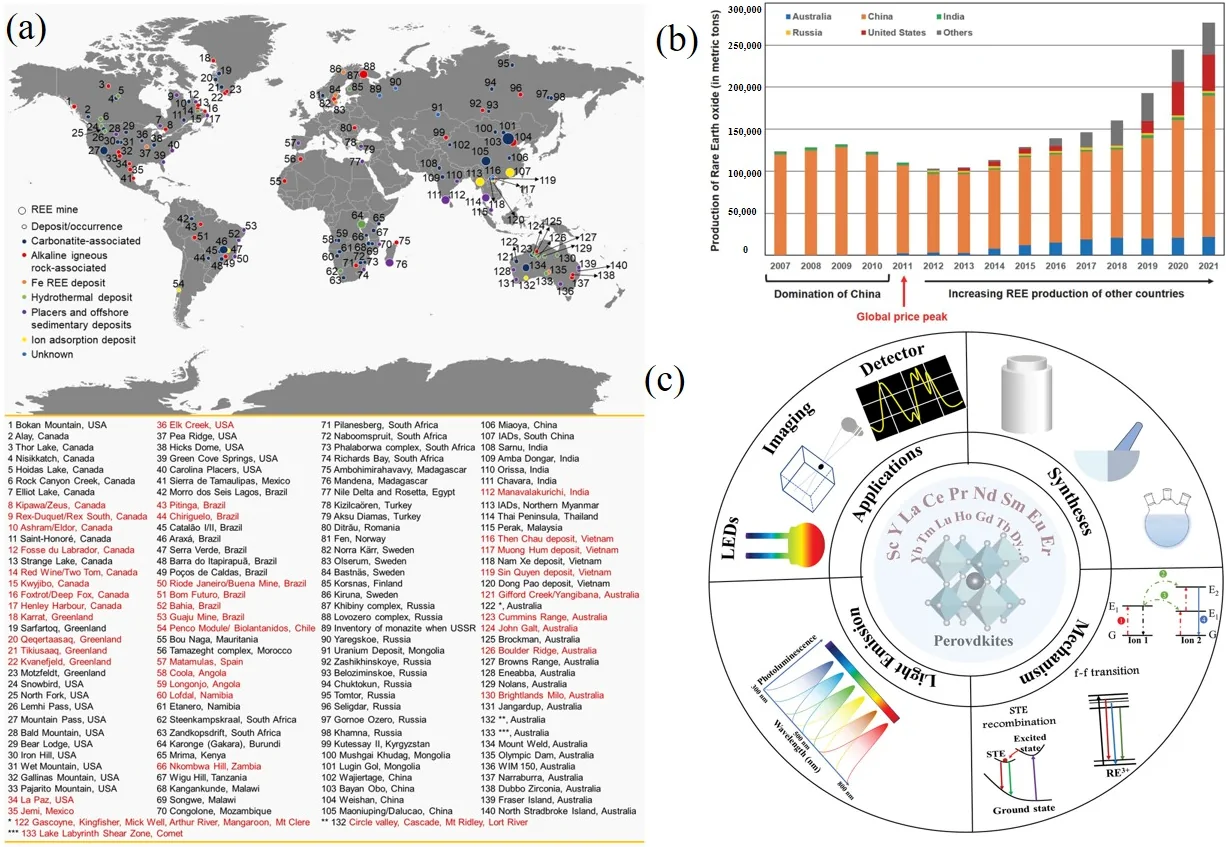
(**a**) Geographic locations of known REE mines and major deposits worldwide [4]. (**b**) Annual global production of rare earth elements over the period 2007–2021 [5]. (**c**) Overview of key end-use applications for rare earth elements [10].

The rare earth elements (REEs) comprise the 15 lanthanides (La–Lu) plus scandium (Sc) and yttrium (Y), totaling 17 elements with similar chemical properties [1,2]. Based on their physicochemical characteristics and resource distribution, REEs are commonly classified into light REEs (LREEs, La-Eu), medium REEs (MREEs, Gd-Ho), and heavy REEs (HREEs, Er-Lu+Y+Sc). Although not particularly scarce in the Earth’s crust, economically viable REE mineralization is strictly limited to specific tectonic settings and magmatic–hydrothermal processes, leading to a markedly uneven global distribution (Figure 1a) [3,4]. China hosts world-class deposits, notably Bayan Obo in Inner Mongolia and Maoniuping in Sichuan, and consequently leads the world in reserves, annual production, and refining capacity. Significant resources are also found in the United States (Mountain Pass), Australia (Lynas), Russia, Brazil, and Vietnam. This pronounced geographic concentration has fundamentally shaped the long-term trajectory of global REE supply. Figure 1b depicts global rare earth production from 2007 to 2021 [5]; for most of this period, China accounted for over 90% of worldwide output, reflecting a highly monopolistic supply structure. Such dominance is not merely a matter of volume. It is reinforced by China’s longstanding command of technologies for high-purity separation, which are especially critical for HREEs, the scarcest and most valuable subset of the REE family [6,7].

From a strategic standpoint, REEs are vital raw materials for permanent magnets; lasers; telecommunications; radar; and clean energy equipment, including wind turbines and electric vehicles; and have been designated as critical minerals worldwide [8,9]. The scarce heavy REEs, combined with China’s dominant refining expertise, render the REE supply chain a key geo-economic and technological battleground. Stable, diversified REE supplies underpin advanced manufacturing independence, national defense reliability and viable low-carbon shifts. In terms of end-use applications, REEs are ubiquitously employed in permanent magnet materials, catalysts, phosphors, hydrogen storage alloys, electronic devices, aerospace components, new energy vehicles, and wind power generation. Often referred to as “industrial vitamins”, they underpin nearly every facet of modern technology. Their detailed application breakdown is provided in Figure 1c [10]. Consequently, ensuring both the supply security and efficient utilization of REEs has become a core challenge intersecting global technological rivalries, industrial policy, and international cooperation [11,12].

### 1.2. Conventional Processes for Rare Earth Extraction and Separation

The extraction and separation of REEs begin with beneficiation of REE-bearing ores, which are first subjected to mining, crushing and physical concentration [13,14]. The beneficiated material is then chemically leached using acidic solutions or salt-based lixiviants to transfer the REEs into an aqueous phase [15,16]. This is followed by solvent extraction, which typically employs organophosphorus extractants, to achieve both group separation and individual purification of REEs [17]. After extraction, stripping is performed to obtain single rare earth chloride solutions. Subsequently, oxalic acid or ammonium bicarbonate is introduced as a precipitant to convert the REEs into insoluble rare earth salts, which are finally calcined at high temperatures to yield high-purity rare earth oxides. The entire production chain must be integrated with auxiliary systems for treating acidic and alkaline wastewater to meet environmental compliance [18].

Among the available separation technologies, solvent extraction stands as the most mature and industrially dominant method, honed over decades of development. Commonly used organophosphorus extractants include P204, P507 and Cyanex272 [19,20]. Specifically, a P507 system is currently the preferred choice for separating LREEs, whereas Cyanex272 exhibits superior selectivity for MREEs and HREEs. Solvent extraction offers significant advantages of high throughput, continuous countercurrent operation, and compatibility with large-scale industrial production. Nevertheless, its intrinsic limitations are equally pronounced [21,22]. First, the separation factors between adjacent REE pairs, such as Pr/Nd and Dy/Ho, are inherently low. This necessitates cascading dozens or even hundreds of extraction stages in series, which leads to bulky equipment, extensive plant footprints, and prohibitively high capital expenditures [23]. Second, the process consumes enormous quantities of organic solvents and kerosene diluents, with substantial evaporative losses occurring during operation. The system is inherently flammable and explosive, presenting serious environmental pollution risks and occupational safety hazards. Furthermore, the extractants are susceptible to degradation and emulsification over time, which increases reagent consumption and also compromises process stability [24,25].

For applications demanding exceptionally high purity, ion exchange and solvent-impregnated resin methods can achieve product purities exceeding 99.99%, making them well suited for trace REE enrichment and preparation of high-purity reagents [26,27]. However, these techniques suffer from critical bottlenecks: a low resin exchange capacity and sluggish mass transfer kinetics result in extremely slow processing rates, and treating just one ton of feed solution often requires several days. In addition, the resins are prone to fouling by impurities and are costly to regenerate, rendering these methods economically unviable for meeting the demands of industrial production at the hundred-thousand-ton scale [28].

Alternatively, chemical precipitation using oxalic acid or carbonates is straightforward in operation and low in cost [29]. Yet this approach is plagued by poor selectivity and severe co-precipitation, making it difficult to achieve the effective fractional separation of adjacent REEs. The purity of the final product typically falls below 95%, and the precipitates tend to be gelatinous and notoriously difficult to filter [30]. Consequently, multiple recrystallization steps are often required to improve product quality, which prolongs the overall processing time and drastically reduces the overall yield. In the face of increasingly stringent environmental regulations and the global push toward green manufacturing, conventional REE separation processes are under enormous pressure to undergo technological transformation [31]. There is, therefore, a compelling need to develop next-generation separation technologies that feature low energy consumption, minimal pollution, high separation efficiency, and superior selectivity.

### 1.3. Emergence of Membrane Separation Technology in Hydrometallurgy

Membrane separation technologies exploit selective permeation, driven by either pressure gradients or electric fields, to fractionate multi-component mixtures [32,33]. Based on their decreasing pore size, pressure-driven membranes are classified as microfiltration (MF, 0.1–10 μm) for suspended particles and bacteria; ultrafiltration (UF, 1–100 nm) for macromolecular proteins and colloids; nanofiltration (NF, ~1 nm) for small organics and multivalent ions permeating monovalent ions; and reverse osmosis (RO, <1 nm), which retains nearly all solutes for seawater desalination [34,35]. Electrodialysis (ED), in contrast, employs an electric field to drive ionic migration through ion exchange membranes and is widely used for desalination and acid/base recovery. Collectively, these membrane processes span a continuous spectrum from coarse particle removal to ion-level fractionation, with progressively enhanced selectivity [36,37].

Among them, NF stands out due to its unique synergistic mechanism combining size sieving with the Donnan (charge) effect, arising from a membrane’s surface charge and nanoscale pore size [38,39]. NF operates at relatively low pressures (typically 0.5–2.0 MPa), similar to UF, yet achieves a solute rejection comparable to RO for solutes with low molecular weights [40,41]. Its distinguishing advantage lies in its valence-based selectivity: monovalent ions (e.g., Na^+^, K^+^) are poorly retained, whereas multivalent ions (e.g., Mg^2+^, Ca^2+^, SO_4_^2−^) and organic molecules in the 200–1000 Da range are rejected at rates exceeding 90%. This characteristic makes NF indispensable in water softening, decolorization, and antibiotic concentration, filling the technical gap where UF leaves impurities and RO incurs excessive energy costs [42,43].

In REE processing, conventional solvent extraction (SX), though mature, suffers from massive consumption of organic solvents and acids/bases, generation of high-salinity wastewater, and operation in batch mode that requires frequent phase transfers [44,45]. These drawbacks are inherent to P507/P204-based SX systems, as discussed earlier. Against this backdrop, NF application to REE separation aligns well with green chemistry principles: (i) low energy consumption, as NF operates at only 0.5–2.0 MPa, far below RO and evaporative concentration, thus significantly reducing the carbon footprint; (ii) no phase transfer, as the entire process remains in a single aqueous phase, eliminating the need for organic extractants, stripping steps, and associated solvent evaporation or entrainment losses; and (iii) environmental benignity—acid, base and flammable organics use is greatly reduced, and the resulting wastewater is simpler to treat [46,47]. NF can also selectively remove impurity cations (e.g., Ca^2+^, Mg^2+^) or pre-fractionate REE ions of different valences (e.g., trivalent vs. divalent) based on size/charge effects. Consequently, NF offers a novel pathway for REE hydrometallurgy that is energy-efficient, clean and readily amenable to continuous automated operation, addressing the structural deficiencies of conventional SX processes [48].

### 1.4. Purpose and Structure of This Review

This review systematically summarizes the research progress and development trends in NF-based separation of REEs. It aims to elucidate the separation mechanisms, advances in membrane materials, influencing factors, application status and techno-economic aspects. It also clarifies the key challenges currently faced. Future directions are proposed to offer a comprehensive reference for advancing the industrial application of NF technology to the green separation and high-value utilization of REEs.

## 2. Fundamental Principles of NF Separation of REEs

### 2.1. Typical Characteristics of NF Membranes

NF membranes feature nominal pore sizes of 0.5–2 nm, corresponding to an MWCO of 200–1000 Da [49]. These nanoscale channels retain multivalent ions and small-to-medium organic molecules, while allowing water, monovalent ions, and low-molecular-weight neutral solutes to permeate at low resistance [50,51]. Most commercial NF membranes are thin-film composite structures with a selective layer bearing functional groups, such as carboxyl, sulfonic, or amine moieties [52]. Upon hydration, these groups dissociate, imparting a surface charge (positive or negative) to the membrane. This charge gives rise to the Donnan (charge exclusion) effect, which influences the transport of ionic species [53]. The membrane’s charge density plays a key role: a higher charge density enhances the rejection of multivalent ions, even at reduced operating pressures. Crucially, the effective size of ions in a solution is their hydrated (Stokes) radius, which determines the steric hindrance during pore passage [49]. Overall, NF separation performance stems from the synergistic combination of size sieving (based on hydrated radius and MWCO) and Donnan exclusion (based on surface charge and ionic valence) [54]. This dual mechanism, integrating steric and electrostatic effects, is the key feature distinguishing NF from other pressure-driven membrane processes.

### 2.2. Hydration Chemistry of Rare Earth Ions

**Figure 2:**
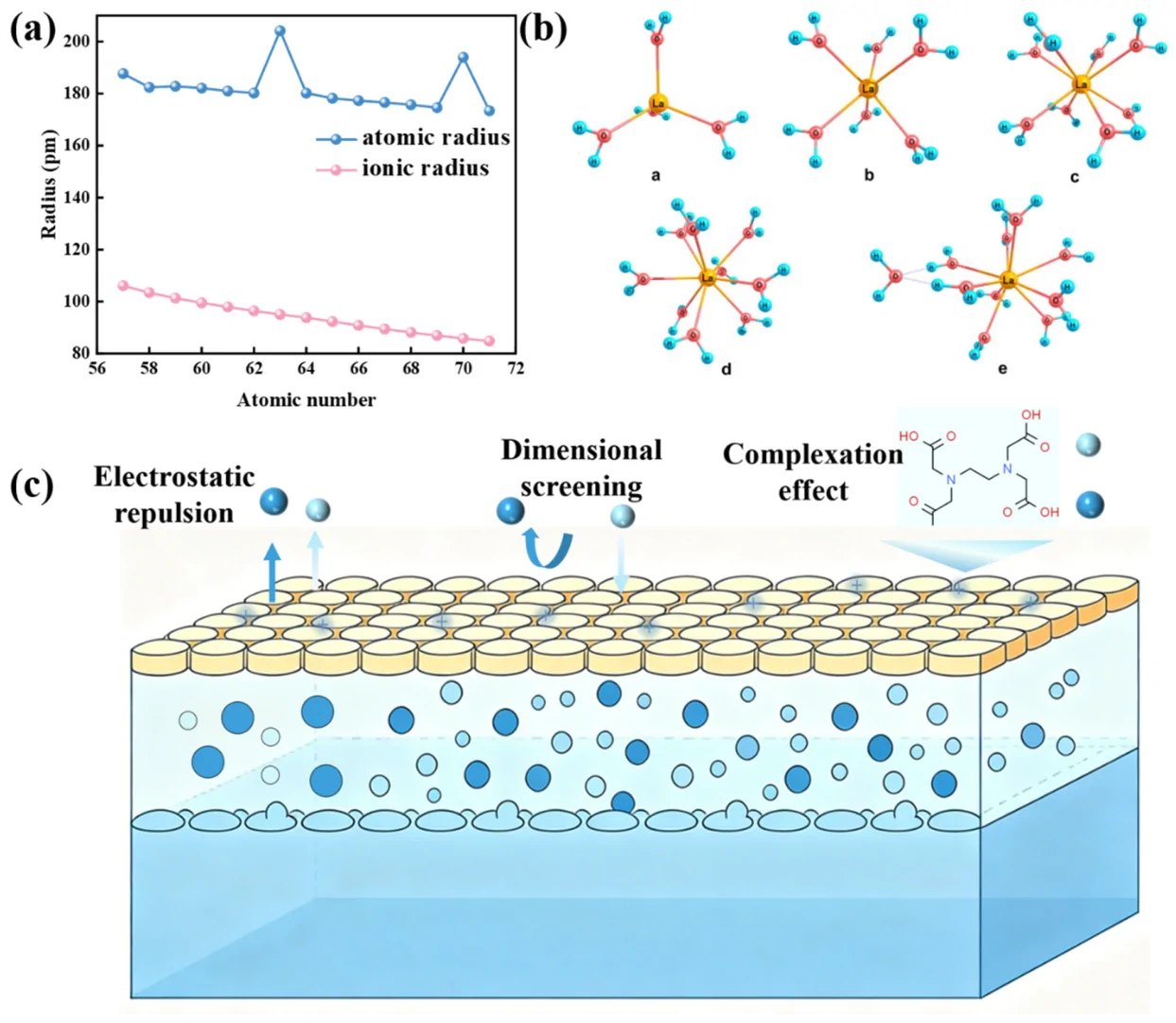
(**a**) The atomic radius and ionic radius of lanthanide elements decrease with an increase in the atomic number; (**b**) spatial models of different hydration numbers of La [59]; (**c**) multiple separation mechanism diagram.

In an aqueous solution, trivalent rare earth ions (Ln^3+^) are strongly hydrated, forming stable [Ln(H_2_O)_n_]^3+^ complexes. This hydration behavior critically governs ion mass transfer and rejection during membrane separation [55,56]. Hydrated (Stokes) radii exhibit a trend opposite to that of crystal radii: light REEs (e.g., La^3+^, Ce^3+^) possess larger hydrated radii, whereas heavy REEs (e.g., Lu^3+^) have smaller ones [57]. The monotonic decrease in ionic radius across the lanthanide series is illustrated in Figure 2a and Table 1. This reversal arises because the lanthanide contraction increases the charge density of the ions, enhancing their polarizing power and electrostatic attraction toward the surrounding water dipoles. This results in a more compact and tightly bound hydration shell, and consequently a smaller effective hydrodynamic radius with an increasing atomic number [58]. This intrinsic size variation provides NF membranes with a potential size-sieving basis for discriminating between adjacent REEs. To further elucidate the underlying hydration structures and binding characteristics, Takayuki et al. [59] systematically investigated La^3+^, Gd^3+^, Y^3+^, and Lu^3+^ using quantum chemical calculations (HF/MP2) and first-principles molecular dynamics simulations. Their results demonstrate that the M-O bond length, which directly reflects the effective hydrated ion size, decreases monotonically as the ionic radius decreases, from 2.60 Å for La^3+^ to 2.36 Å for Lu^3+^, quantitatively manifesting the lanthanide contraction in a hydrated state. For a given ion, increasing the coordination number from eight to nine enlarges the hydrated ion by approximately 0.3 Å, while the binding energy per bond decreases with a rising coordination number. The magnitude of this decrease follows the order La^3+^ < Gd^3+^ < Y^3+^ < Lu^3+^, inversely correlating with the ionic size. Figure 2b presents the optimized geometries of La(H_2_O)_n_^3+^ for n = 4, 6, 8, 9, and 10, revealing a coordination number transition from nine (for light La^3+^) to eight (for heavier Y^3+^ and Lu^3+^), consistent with the decreasing ionic radius. Notably, no distinct “gadolinium break” discontinuity is observed in this coordination transition.

Beyond pure hydration, REE speciation in real processing solutions is highly sensitive to the pH and ligand chemistry [60,61]. Under acidic conditions, Ln^3+^ remains the dominant free ion. However, as the pH rises (typically > 5–6), hydrolysis progressively occurs, forming mononuclear hydroxo complexes, such as Ln(OH)^2+^ and Ln(OH)_2_^+^, which carry reduced positive charges and undergo further aggregation into polynuclear species at elevated concentrations. In the presence of carbonate/bicarbonate (common in natural waters or certain leach liquors), stable anionic carbonato complexes, such as Ln(CO_3_)_2_^−^ and Ln(CO_3_)_3_^3−^, can form, effectively converting the cationic Ln^3+^ into negatively charged species [62]. These transformations induced by the pH and ligands dramatically alter the effective charge, hydrated size, and diffusion coefficient of the REE-containing solutes, thereby profoundly affecting their electrostatic and steric interactions with the charged NF membranes [63]. Consequently, a thorough understanding of REE hydrolysis and complexation equilibria is indispensable for accurately predicting and optimizing NF-based separation performance under realistic process conditions. Taken together, the experimental and computational evidence confirms that the size, binding strength, coordination number, and speciation of rare earth hydrated ions are intrinsically governed by the lanthanide contraction, with superimposed modulations by the solution chemistry [64]. This hierarchical understanding provides a solid physicochemical foundation for designing membrane-based REE separation strategies.

**Table 1:** Various relevant information on lanthanide elements and Sc and Y [65].

Element	Atomic Number	Atomic Radius (pm)	Ionic Radius (M^3+^, pm)	Symbol
Lanthanum	57	187.7	106.1	La
Cerium	58	182.4	103.4	Ce
Praseodymium	59	182.8	101.3	Pr
Neodymium	60	182.1	99.5	Nd
Promethium	61	181.0	97.9	Pm
Samarium	62	180.2	96.4	Sm
Europium	63	204.2	95.0	Eu
Gadolinium	64	180.2	93.8	Gd
Terbium	65	178.2	92.3	Tb
Dysprosium	66	177.3	90.8	Dy
Holmium	67	176.6	89.4	Ho
Erbium	68	175.7	88.1	Er
Thulium	69	174.6	86.9	Tm
Ytterbium	70	194.0	85.8	Yb
Lutetium	71	173.4	84.8	Lu
Scandium	21	164.1	68.0	Sc
Yttrium	39	180.1	88.0	Y

### 2.3. Synergistic Mechanisms of NF Separation of REEs

The nanofiltration separation of REEs is governed by a synergistic interplay of size exclusion, electrostatic repulsion, dielectric exclusion, and complexation enhancement [66]. As illustrated in Figure 2c, coupling these mechanisms can markedly improve separation selectivity beyond the intrinsic membrane sieving capability. (1) Size exclusion exploits the subtle but systematic differences in the hydrated radii across the lanthanide series. LREEs, possessing larger hydrated radii than HREEs, are preferentially retained, whereas HREEs exhibit relatively higher permeation. Although the differences between adjacent REEs are minute (typically < 0.2 Å), they become exploitable when the membrane pore size distribution is tightly controlled and operated near the MWCO threshold [67]. (2) Electrostatic repulsion (Donnan effect) arises from the membrane surface charge interacting with the ionic solutes. On a positively charged NF membrane, trivalent Ln^3+^ ions experience substantially stronger repulsion than monovalent (Na^+^, K^+^) and divalent (Ca^2+^, Mg^2+^) species, enabling the efficient decontamination of REEs from alkali and alkaline earth impurities. The rejection efficiency scales with the ionic valence and membrane charge density [68]. (3) Dielectric exclusion originates from the energy barrier encountered when ions transfer from a high-dielectric aqueous phase into a low-dielectric membrane polymer matrix. This energetic penalty is considerably larger for multivalent ions than for monovalent ones, thus providing an additional repulsive contribution that becomes particularly significant in high-salinity or concentrated feed solutions [69]. (4) Complexation-enhanced separation involves the addition of complexing agents (e.g., EDTA, DTPA, citric acid, NTA) to the feed, which form stable REE complexes with substantially different sizes, charges, and steric configurations compared to free hydrated ions [70]. The differences among these complexed species are far greater than those among free Ln^3+^ ions, thereby dramatically amplifying the separation factors and enabling the efficient discrimination of adjacent REEs, an approach termed complexation nanofiltration (CNF).

In the absence of complexing agents, the three purely physicochemical mechanisms, namely size exclusion, electrostatic repulsion, and dielectric exclusion, operate concurrently to achieve preliminary REE fractionation. Among these, electrostatic repulsion plays the dominant role: rejection of Ln^3+^ correlates directly with the membrane surface charge density. The higher the negative (or positive) charge, the more pronounced the retention. However, as the ionic strength increases (which is the case for real REE leachates, I ≈ 0.5–2.0 M), counterion adsorption and electrical double-layer compression are aggravated, severely weakening the Donnan exclusion. Recent quantitative studies have revealed that under high-salinity conditions, dielectric exclusion contributes approximately 75% to the rejection of multivalent anions (e.g., SO_4_^2−^), whereas the contribution of Donnan exclusion is fairly moderate, highlighting the critical role of pore size-influenced dielectric effects in ion-selective separation [35]. Dielectric exclusion, though weaker in magnitude, retains a non-negligible rejection contribution in high-salinity systems where Debye screening partially attenuates the electrostatic effects, thereby serving as an important auxiliary mechanism. Size exclusion, meanwhile, provides the baseline selectivity but is inherently limited by the small differences in adjacent REE hydrated radii. Complexation enhancement represents the critical breakthrough pathway toward highly selective separation of adjacent REEs. Complexing agents exhibit distinct stability constants with individual REEs, allowing for the precise modulation of complex charge, size and transport properties. For instance, DTPA forms complexes with HREEs whose stability constants are 1–2 orders of magnitude higher than those with LREEs; the resulting HREE complexes carry higher negative charges and larger hydrodynamic dimensions, leading to a markedly elevated rejection of HREEs relative to LREEs, thereby effectively reversing the native size exclusion order. Citric acid, as a green complexant, is nontoxic and readily biodegradable, making it attractive for industrial implementation. Under mildly acidic conditions (pH 5–6), its selective complexation toward REEs is optimized, yielding separation factors that increase by more than an order of magnitude compared to ligand-free operation. Crucially, the synergy among these four mechanisms upgrades NF separation from simple physical sieving to a precision recognition process. By rationally tailoring the membrane properties (pore size, charge density) and solution chemistry (ligand type, pH, ionic strength), the inherent trade-off between permeability and selectivity can be partially circumvented [71].

### 2.4. Key Parameters Influencing Separation Performance

NF’s separation performance for REEs, including the permeate flux, rejection efficiency, separation factor, and long-term stability, is co-determined by the operating parameters and solution chemistry, whose effects are often coupled rather than independent. The operating conditions exert direct and sometimes nonlinear influences on the NF performance. (i) The transmembrane pressure increases the permeate flux proportionally within a pressure-dependent regime. Beyond a critical threshold, the enhancement diminishes as the concentration polarization intensifies, leading to a plateau or even a decline in rejection due to enhanced solute back diffusion and elevated wall concentration [72]. (ii) The cross-flow velocity mitigates the concentration polarization by reducing the boundary layer thickness at the membrane surface, improving both the flux stability and rejection consistency. This benefit comes at the cost of elevated pumping energy consumption [73]. (iii) The feed temperature reduces solution viscosity, enhancing diffusivity and thus permeate flux. Temperature elevation also modifies the membrane pore dimensions via thermal expansion and may alter the ion hydration shell dynamics, potentially compromising rejection selectivity, particularly when structural changes in the polymer separation layer occur [74].

The solution chemistry introduces equally profound, and often more complex, effects. (i) The pH exerts a dual influence: it governs the protonation state of membrane surface functional groups (hence the charge density) and dictates REE speciation. Under acidic conditions (pH < 4), REEs persist as free Ln^3+^ cations, experiencing strong electrostatic repulsion from positively charged membranes, and yielding a high rejection. Near neutrality (pH 5–7), hydrolysis progressively generates hydroxo complexes (e.g., Ln(OH)^2+^, Ln(OH)_2_^+^) with a reduced charge, lowering rejection; a further pH increase may induce precipitation or polynuclear species formation, complicating separation. (ii) The ionic strength modulates the Debye screening length: an elevated salt concentration compresses the electrical double layer, weakening the Donnan exclusion and consequently reducing multivalent ion rejection, a phenomenon particularly relevant in high-salinity leach liquors. (iii) Coexisting ions, especially multivalent cations such as Al^3+^ and Fe^3+^, not only compete with Ln^3+^ for membrane surface charge sites, attenuating electrostatic repulsion, but also readily hydrolyze to form colloidal hydroxides, which deposit on the membrane surface as foulants, accelerating flux decline and compromising separation stability. (iv) The feed concentration directly intensifies the concentration polarization and fouling propensity: as the REE concentration rises, the permeate flux declines monotonically, and the separation factor for adjacent REE pairs generally deteriorates due to increased solute–solute interactions and enhanced coupled transport [75,76].

Crucially, these parameters do not operate in isolation. For instance, the pH-dependent charge density modulates the sensitivity of rejection to the ionic strength. An elevated temperature can exacerbate fouling by accelerating precipitation of sparingly soluble species, and a higher cross-flow may partially offset the adverse effects of high feed concentration [77]. Therefore, the rational optimization of REE NF separation demands a holistic, multi-parameter approach that balances productivity, selectivity, and energy consumption, while being tailored to the specific feed composition and target separation objectives.

## 3. NF Membrane Materials and Modifications for REE Separation

### 3.1. Application of Commercial NF Membranes in REE Separation

Commercial NF membranes (e.g., NF270, NF90) benefit from mature fabrication technologies and controllable costs; they can effectively reject REE ions via size sieving and charge exclusion, facilitating their large-scale application. Table 2 lists the commonly used commercial NF membranes. Despite this potential, their limitations remain prominent. Insufficient selectivity is the primary bottleneck: these membranes mainly rely on size sieving and charge exclusion to separate ions of different valences, but for isovalent lanthanides (e.g., Nd^3+^ and Er^3+^), the difference in hydrated ionic radius is merely ~0.02–0.03 nm, far smaller than the pore size distribution of NF membranes. Consequently, the separation factor between adjacent REEs generally does not exceed two, which fails to meet the demands of high-purity separation. These inherent shortcomings have motivated extensive research into two complementary avenues: modifying conventional polymeric NF membranes to enhance their intrinsic selectivity and exploring new membrane architectures that go beyond classic polyamide chemistry [78].

### 3.2. Modification Strategies for Polymeric NF Membranes

To overcome the performance bottlenecks of conventional commercial NF membranes, particularly selectivity, researchers have adopted three main strategies: optimization of interfacial polymerization (IP) conditions, surface grafting and coating, and incorporation of nanofillers [85]. Among these, IP condition optimization is the most straightforward approach, as it enables the direct tuning of the separation layer microstructure. By finely adjusting parameters such as monomer concentration, reaction time and heat treatment, researchers have effectively improved both the separation precision and permeate flux. For instance, Sun et al. [86] used piperazine (PIP) and toluene diisocyanate (TDI) as monomers to prepare a polyurea NF membrane via IP, and optimized the monomer concentration and reaction time using response surface methodology. The resulting membrane exhibited a water flux of 21.1 L·m^−2^·h^−1^ at 15.5 bar. When treating simulated NdFeB acidic wastewater, it achieved over 97% rejection for Nd^3+^, Pr^3+^, Dy^3+^ and Co^2+^, demonstrating a robust sieving capability for individual REE ions.

A second strategy, surface grafting and coating, involves post-modification of the separation layer to regulate the surface charge, hydrophilicity, and antifouling properties [87]. While IP optimization enhances selectivity primarily by refining the pore structure, grafting and coating tackle the same challenge through complementary electrostatic interactions. Common approaches include chemical grafting of charged polymers or physical deposition of functional coatings. Unlike IP optimization, which primarily alters the pore size, grafting/coating directly modulates the membrane’s electrostatic interactions with solutes. Léniz Pizarro et al. [80] introduced poly(allylamine hydrochloride) (PAH) as a grafting component into a PIP system to fabricate a positively charged NF membrane (PIP/PAH PS35). This membrane showed rejection rates of approximately 99.3% and 98.5% for 1 mM LaCl_3_ and NdCl_3_, respectively, while NaCl rejection was only 74.5% (with a flux of ~34 L·m^−2^·h^−1^). In a mixed feed containing 100 mM NaCl and 1 mM LaCl_3_, the La^3+^ rejection remained stably above 99%, and the Na^+^/La^3+^ selectivity factor reached 22.5, indicating excellent stability and REE selectivity and providing a feasible route for efficient REE recovery from high-salinity streams.

**Figure 3:**
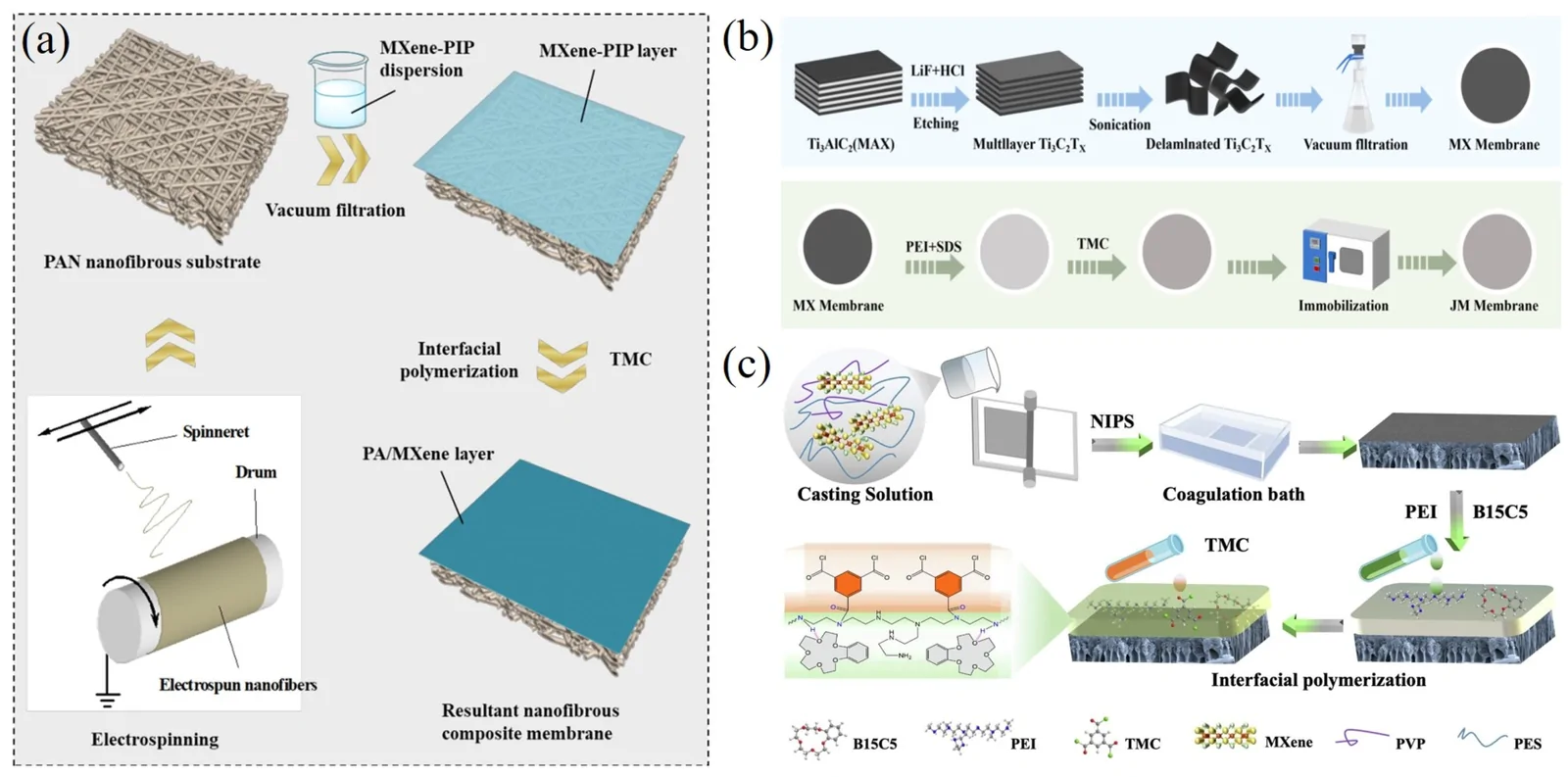
Regulation of nanofiltration membrane preparation by MXene at different positions: (**a**) polyamide layer [89], (**b**) intermediate layer [90], (**c**) substrate layer [91].

The third strategy, incorporating nanofillers, offers tunability because the filler location, whether within the polyamide layer, as an intermediate layer, or blended in the substrate, fundamentally alters the separation mechanism [88]. Taking two-dimensional MXene as an example, Zhang et al. [89] employed a vacuum-assisted co-deposition method to mix MXene nanosheets with PIP and coated the mixture onto an electrospun PAN substrate, followed by IP to prepare a composite NF membrane (Figure 3a). The membrane achieved a Na_2_SO_4_ rejection of 97.2% and a pure water flux of 152.4 L·m^−2^·h^−1^ at 0.6 MPa, demonstrating significant enhancement of permeability–selectivity by embedding MXene within the PA layer. Bao et al. [90] used negatively charged MXene as an intermediate layer and then polymerized a positively charged PA layer on top, successfully fabricating an MXene–PA bilayer Janus NF membrane (Figure 3b). At 0.6 MPa, this membrane exhibited rejections of 92% for La^3+^, 94% for Y^3+^, and 94.25% for Gd^3+^, with a stable water flux of 15–20 L·m^−2^·h^−1^. Wang et al. [91] directly added MXene during the phase inversion process to prepare a B15C5-MX-NF membrane (Figure 3c), which achieved a pure water permeance approximately double that of the unmodified membrane, reaching 16.1 L·m^−2^·h^−1^·bar^−1^. Collectively, these studies demonstrate that nanofiller positioning strategies can enhance membrane performance from different perspectives, providing multiple design inspirations for developing high-performance REE-selective NF membranes.

Despite the impressive performance enhancements achieved by these two-dimensional nanomaterials, their structural stability during interfacial polymerization remains a critical concern. The harsh chemical environment of the IP process, involving reactive monomers (e.g., PIP, TMC), organic solvents, and acidic/basic conditions, can induce nanofiller aggregation, interfacial incompatibility, or even chemical degradation of the modifiers, compromising the formation of defect-free selective layers and undermining the long-term separation performance. For instance, MXene nanosheets with abundant hydrophilic terminations are prone to restacking and oxidation when exposed to an aqueous–organic interface, leading to non-uniform dispersion within a polyamide matrix. Ensuring the robust stability of 2D nanofillers under IP conditions is a prerequisite for translating laboratory-scale membrane modifications into reliable and scalable REE separation applications.

Beyond MXene, a wide range of other nanomaterials have also been extensively explored for mixed matrix membrane (MMM) fabrication. Yu et al. [92] embedded oxidized graphitic carbon nitride (OGCN) nanoparticles into PEI via immersion precipitation phase inversion, followed by interfacial polymerization with PIP and TMC. The resulting membrane exhibited a pure water flux of 38.6 L·m^−2^·h^−1^ at 0.6 MPa, with rejections for La^3+^, Y^3+^, and Nd^3+^ reaching 95.8%, 96.7%, and 95.3%, respectively. At a volume concentration factor of 10, the REE concentration increased from 0.242 g/L to 1.285 g/L, while REE loss in the permeate was controlled within 65 mg, significantly lower than the 80 mg of the unmodified membrane. Moreover, the rejection remained stable at approximately 97% during continuous operation for 1440 min, with only a slow flux decline. Jiang et al. [93] embedded O-MoS_2_ nanosheets into a PSF substrate via non-solvent-induced phase inversion to form a nanocomposite UF support layer, followed by reaction of PIP with TMC. The optimized membrane achieved a salt rejection of 97.08% and a flux of 30.8 L·m^−2^·h^−1^ in simulated REE wastewater containing 2 g/L Na_2_SO_4_ and 0.001 g/L P507 surfactant, representing a significant improvement over the unmodified membrane. During 24 h continuous operation with real acidic REE wastewater (pH 2.46), the salt rejection remained stable above 95%, with only a 12.8% decline in flux.

Collectively, these studies demonstrate that nanomaterials synergistically enhance membrane selectivity toward REE ions through two complementary effects: first, the abundant functional groups containing oxygen (e.g., hydroxyl, carboxyl) on their surfaces act as specific adsorption sites, preferentially coordinating with or electrostatically binding trivalent REE ions; second, the nanofillers themselves strengthen the size-sieving effect by creating additional nanochannels or altering the membrane pore architecture. This dual mechanism, which combines enhanced affinity and refined steric exclusion, effectively boosts the separation factors beyond those achievable by pristine polymeric membranes. The underlying mechanisms differ markedly with the filler location: embedding within the PA layer introduces additional nanochannels and charge sites that enhance ion selectivity in synergy; interlayer construction retards aqueous-phase monomer diffusion during IP, yielding a thinner, more permeable PA layer; and substrate blending improves the base membrane’s hydrophilicity and pore structure, reducing transmembrane resistance. Each approach addresses a different level of the membrane hierarchy, and their combined insights guide rational design for specific REE separation targets.

### 3.3. Novel NF Membrane Architectures

**Figure 4:**
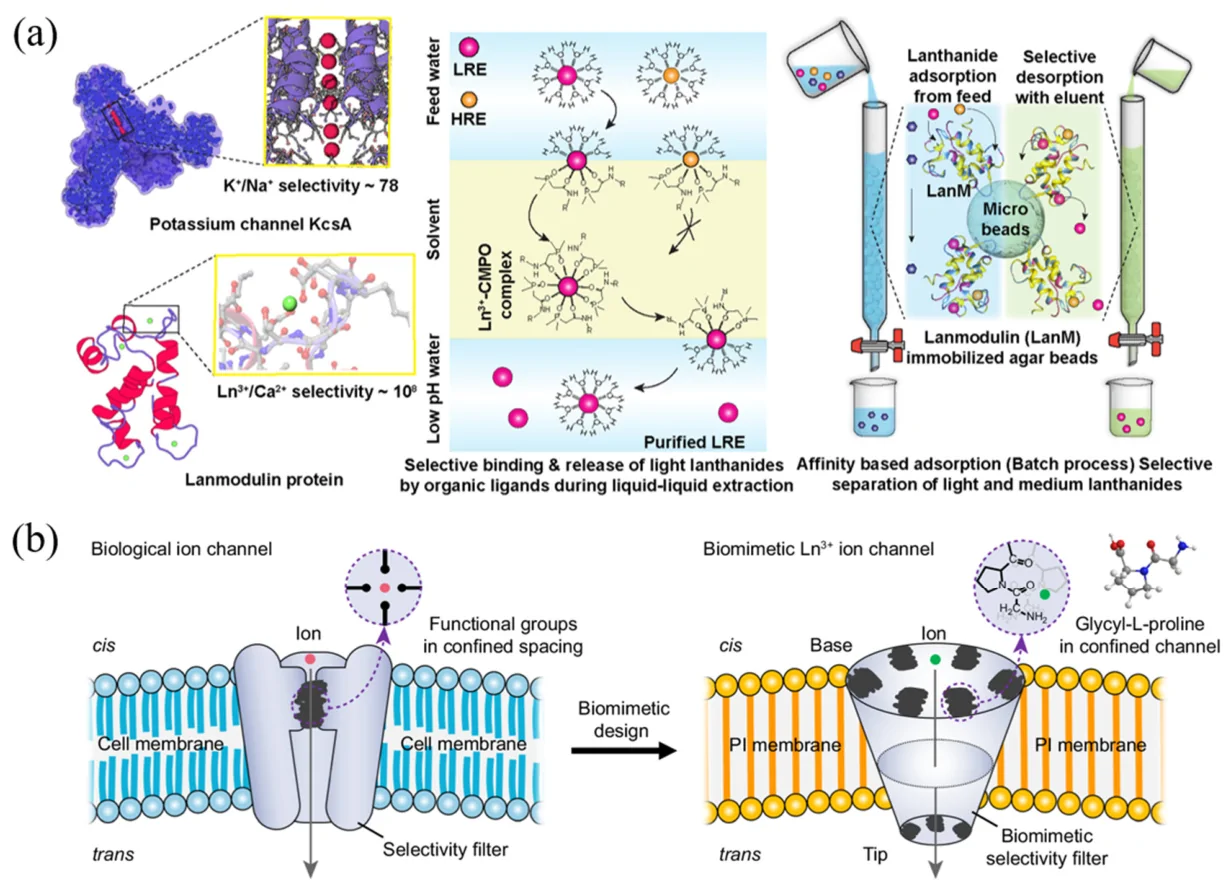
Natural biological ion-selective units and the design of biomimetic rare earth separation materials. (**a**) High-selectivity recognition mechanisms of KcsA potassium channel and lanthanide- binding Lanmodulin, as well as rare earth separation processes via liquid–liquid extraction and protein affinity chromatography [97]. (**b**) Biomimetic polyimide rare earth sieving channel membrane modified with glycyl-L-proline derived from cell membrane ion channels [98].

While polymeric modification strategies have considerably improved the valence-based selectivity of NF membranes, they still struggle to differentiate adjacent lanthanides with nearly identical hydrated radii. This intrinsic limitation has prompted a more radical departure: mimicking biological ion channels, which achieve extremely high selectivity through precise steric and chemical recognition [94,95]. Biomimetic NF membranes emulate these natural systems and offer the promise of high selectivity and permeability. For REE separation, they can precisely discriminate between adjacent lanthanide ions, enabling efficient sieving and providing a new pathway for green REE extraction. In early studies, Zhong et al. [96] prepared a biomimetic NF membrane embedded with aquaporin Z on a modified cellulose acetate substrate, verifying the feasibility of biomimetic water channels for efficient water transport and salt rejection under pressure. More recently, targeting the highly challenging separation of REEs, Behera et al. [97] constructed a lanthanide-selective artificial channel membrane by connecting diphenylphosphine oxide ligands through a pillararene scaffold (Figure 4a). This design harnesses subtle differences in hydration dynamics within the channel to achieve preferential transport of middle lanthanides, with a Tb^3+^/La^3+^ selectivity of up to ~140 and an Eu^3+^/La^3+^ selectivity exceeding 40. Xin et al. [98] fabricated a biomimetic nanofluidic channel capable of unidirectional ultrafast transport of dysprosium ions by asymmetric track-etching and glycyl-L-proline modification (Figure 4b). This channel amplified the minute affinity differences arising from the ion radius and coordination matching within nano-confined spaces, achieving a Dy^3+^/Nd^3+^ selectivity of ~60 in mixtures of various Ln^3+^ ions. Remarkably, it could preferentially transport Dy^3+^ from a Nd^3+^ solution containing ultra-low Dy^3+^ (3 wt %), thereby increasing the purity of Nd^3+^ in the receiving solution to 99.8 wt %, demonstrating enormous potential for REE resource recovery and purification.

Despite their exceptional selectivity, biomimetic membranes often involve complex fabrication procedures and limited scalability. An alternative paradigm that does not require elaborate channel construction is the use of stimuli-responsive membranes, which leverage external signals (pH, temperature, or electric field) to dynamically modulate the separation performance. Lopez et al. [79] compared three NF membranes (NF270, Desal DL, HydraCoRe 70 pHT) for treating acid mine drainage (pH 1). NF270 (poly(piperazinamide)) showed > 98% rejection of multivalent metals (including REEs) due to its positive surface charge, while allowing for H^+^ and Cl^−^ permeation and achieving negative H^+^ rejection. The sulfate (mainly HSO_4_^−^) rejection was 52–64%. Increasing the Fe(III) concentration raised the sulfate rejection to ~80% with little effect on the metal rejection. By contrast, the negatively charged HydraCoRe 70 pHT resulted in poor metal rejection and high anion rejection, making it unsuitable for acid recovery. Pramanik et al. [99] extended the operating conditions from single pH to combined pH and temperature control, using commercial polyamide membranes to treat simulated AMD containing La, Ce, and Dy. In the AL-FS mode, Dy rejection decreased from 96% at pH 7 to 84% at pH 3; La and Ce rejections were 91% in AL-FS mode and dropped to 85% in AL-DS mode. When the feed temperature was raised to 40 °C, REE rejection further declined by approximately 5%, with the largest decrease observed under a temperature difference mode (40 °C feed/20 °C draw). Xing et al. [100] achieved a breakthrough in electric field responsiveness by constructing an in situ P204/TBP liquid separation layer within a three-dimensional porous CNT membrane. By coupling the cell voltage with the pulsed potential, the electrochemical redox reaction dynamically regulated the complexation and release of target ions, resulting in a Nd^3+^ mass transfer rate 7.45 times higher than that of Dy^3+^. After five enrichment cycles, the separation factor reached 9.56, thereby elevating the separation driving force from passive dependence on the solution chemistry to active external field regulation.

## 4. Experimental Advances in NF Separation of REEs

### 4.1. NF Behavior in Single REE Solutions

The rejection rates of different REE ions vary significantly, and their order is primarily governed by the hydrated ionic radius. Driven by the lanthanide contraction, the ionic radius decreases with an increasing atomic number. Although the enhanced charge density thickens the hydration shell, the overall hydrated radius still trends downward. When ions pass through membrane pores, steric hindrance weakens as the hydrated radius decreases, so ions with larger hydrated radii achieve higher rejection. In the single salt separation experiments conducted by Zagabathuni [101] using an NF300 membrane, the rejection of Ce^3+^ was 94.7%, surpassing the 92.5% for Nd^3+^, which exactly reflects this regularity. From the perspective of non-sieving mechanisms, the Donnan exclusion effect exhibits a pronounced dependence on the electrolyte valence: rejection increases with the charge of the coion and decreases with the charge of the counterion. These findings elucidate the NF separation patterns of single REE solutions and provide an important reference for the further separation of multi-component systems.

### 4.2. Separation of Binary/Multi-Component Mixed REE Systems

Compared with the separation of single REE systems by NF membranes, the challenges of practical process streams often arise from complex mixed systems containing multiple coexisting elements. REEs, with their similar ionic radii (0.85–1.22 Å) and uniform +3 oxidation state, are regarded as one of the most chemically homogeneous element families. The monotonic change in the hydrated radius caused by lanthanide contraction generates distinguishable differences in the hydrated radii between LREEs (e.g., La^3+^, ~4.52 Å) and HREEs (e.g., Lu^3+^, ~3.95 Å). These differences in the hydrated ion size and dielectric exclusion provide a thermodynamic basis for NF membranes to achieve separation based on size sieving and charge exclusion; yet, their capability boundary quickly becomes apparent when facing isovalent REE ions [102]. To navigate the progressively more demanding challenges as the target ions become chemically similar, this section follows four levels of increasing separation difficulty: (1) valence-based separation of REEs from monovalent/divalent impurities via direct NF; (2) desalination and purification of mixed feeds using NF-DF cascade to create a clean ionic environment for subsequent fractionation; (3) complexation-assisted NF to amplify the physicochemical differences between LREEs and HREEs; and (4) shear-induced dissociation (SID) to break the adjacent REE bottleneck. Each tier builds upon the previous, as direct NF alone proves insufficient for the latter three tasks.

Zhao et al. [81] compared the separation performance of three commercial NF membranes for ions of different valences in REE leachate: NF90, with an excessively small pore size, rejected over 99% of all ions, yielding no separation; NF270, with an appropriate pore size and strong negative charge, rejected only 22.09% of Na^+^ but over 89% of Ca^2+^, Nd^3+^, and Ce^4+^, achieving Na^+^/Ca^2+^, Na^+^/Nd^3+^, and Na^+^/Ce^4+^ separation factors of 7.46, 11.88, and 12.43, respectively, making it suitable for monovalent/multivalent ion separation; while NF5, having larger pores, achieved a Ca^2+^/Nd^3+^ separation factor of 6.70 but a Nd^3+^/Ce^4+^ separation factor of only 1.05, exhibiting virtually no selectivity. These results clearly indicate that direct NF relying solely on membrane pore size and surface charge cannot accomplish the mutual separation of isovalent LREEs and HREEs, and additional chemical regulation must be introduced.

Before carrying out the fine separation of LREEs and HREEs, desalination and purification of the mixed feed are necessary engineering steps. The combined NF-DF process proposed by Wang et al. [103] employs a positively charged membrane in the NF stage to retain and enrich all trivalent lanthanide ions while excluding monovalent interfering species, such as Na^+^, followed by selective elution under acidic conditions in the DF stage. Starting from an equimolar La/Na mixture, only 2.4 diafiltration volumes are required to reduce the proton concentration by 3–4 orders of magnitude, ultimately yielding a La concentrate with a molar purity of 99%, thus creating a clean ionic environment for the subsequent separation of LREEs from HREEs and even adjacent REEs.

**Figure 5:**
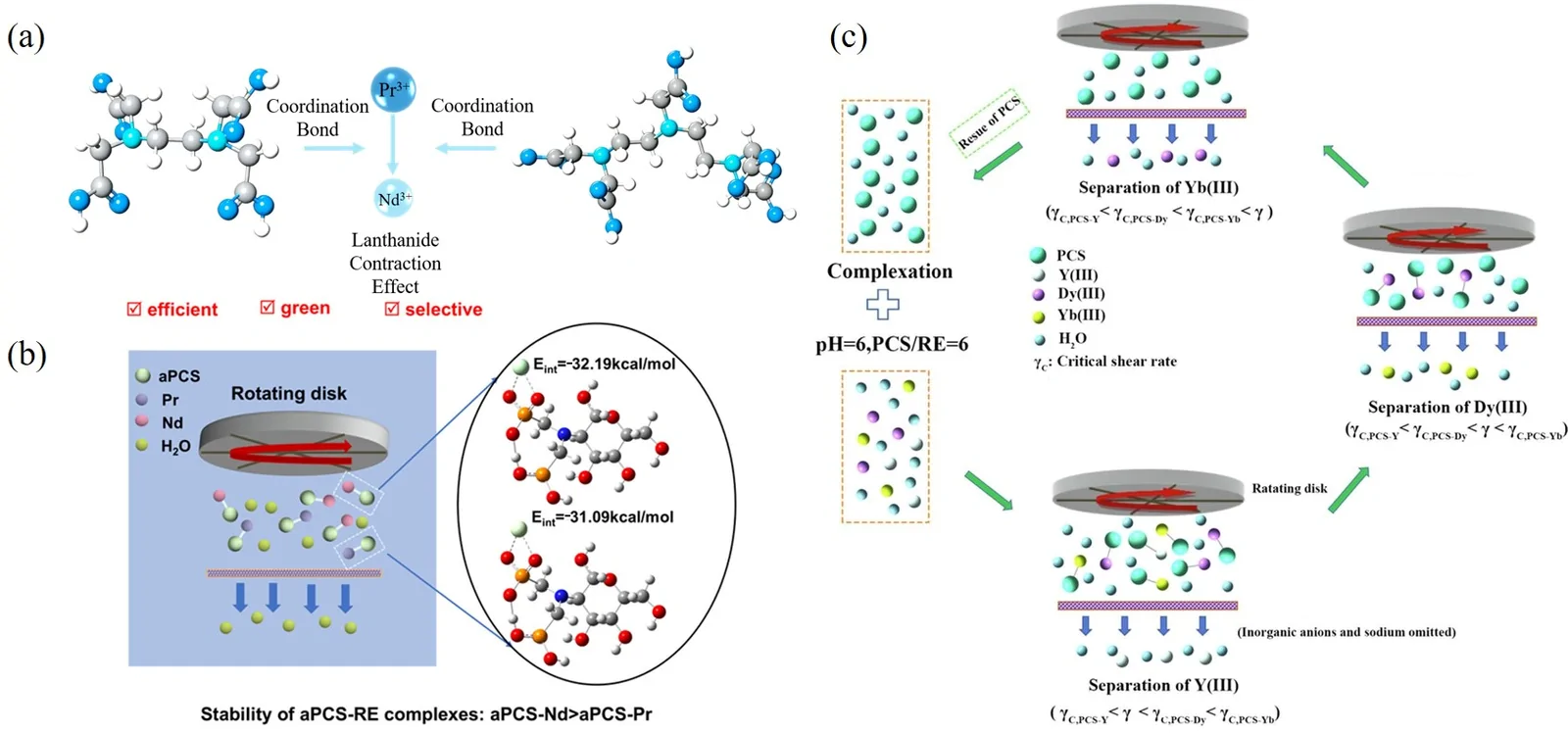
Mechanism of rare earth fractionation via polysaccharide complexation coupled with shear-induced dissociation membrane separation. (**a**) Lanthanide contraction leads to distinct coordination interactions between ligands and Pr^3+^/Nd^3+^. (**b**) Separation of Pr and Nd in a rotating disk shear dissociation membrane system; molecular simulation verifies that the aPCS-Nd complex exhibits higher stability than the aPCS-Pr complex [108]. (**c**) Stepwise membrane separation of Y(III), Dy(III) and Yb(III) based on the discrepancy in the critical shear rates of PCS rare earth complexes [109].

Complexing agent-assisted NF is a key strategy to overcome the limitations of direct NF. The polyaminopolycarboxylic acid ligands EDTA and DTPA are the most extensively studied complexing agents, and the lanthanide contraction is equally significant in complexation behavior. The stability constants (log K) of REE ligand complexes increase monotonically with the atomic number [104,105]. Taking Pr/Nd as an example, the log K values of EDTA with Pr^3+^ and Nd^3+^ are 16.40 and 16.61, respectively, with a difference of only 0.21. For DTPA, the corresponding values are 21.85 and 22.17, with an increased difference of 0.32; the microscopic complexation diagram is shown in Figure 5a. Although the difference remains minute, the higher selectivity of DTPA has already provided exploitable chemical leverage for adjacent REE separation. In the realm of LREE/HREE separation, Borrini et al. [106] employed a tubular TiO_2_/ZrO_2_ inorganic NF membrane with DTPA as the complexing agent to study Eu^3+^/La^3+^ separation. At pH > 2.5, the positively charged membrane rejected over 90% of free Ln^3+^ ions, but upon addition of DTPA, the formed negatively charged complexes (e.g., [EuDTPA]^2−^) were able to permeate the membrane, a mechanism diametrically opposite to steric-based separation. By exploiting the preferential formation of Eu^3+^ complexes at a lower pH, a La^3+^/Eu^3+^ separation factor of 2.5 was obtained at pH 1.9, and using HEDTA, the factor reached 7.5 at pH 2.3, demonstrating that the core driving force of LREE/HREE separation is not size sieving but the electrostatic interaction between the complexes and the positively charged membrane. Also using DTPA, Rahayu et al. [107] prepared Gd-DTPA complexes by reflux reaction of Gd_2_O_3_ with DTPA and then directly separated free Gd^3+^ by NF. As the operating pressure increased from 2 to 6 bar or the feed temperature rose from 25 °C to 40 °C, the permeate flux increased linearly while rejection of free Gd^3+^ continuously declined, exhibiting a typical flux–rejection trade-off and indicating the need for parameter optimization in practical applications of this process.

When the separation target advances to adjacent REEs (e.g., Pr/Nd, Dy/Ho), the challenge escalates further. The ionic radius difference between adjacent lanthanides is typically only ~1 pm, and their hydrated radii almost completely overlap. Neither size sieving nor Donnan exclusion alone can provide sufficient selectivity, necessitating the use of complexation to amplify the physicochemical differences among ions. Within this framework, exploiting the stability differences in complexes under shear fields, namely shear-induced dissociation (SID), has become an emerging strategy, and the related work is shown in Figure 5b,c. Zhou et al. [108] employed acidic phosphorylated chitosan (aPCS) to assist in the membrane separation of Pr/Nd. At pH 5.5, rejections exceeded 97% and the Pr/Nd separation factor reached as high as 12.09. The kinetic studies followed a pseudo-first-order model, with the complexation rate constant of Nd^3+^ being significantly higher than that of Pr^3+^. The DFT calculations confirmed that aPCS-Nd possesses stronger coordination bonds and higher stability. After multiple cycles with the complexing agent, the rejection remained above 90%. Li et al. [109] used phosphorylated chitosan (PCS) as the complexing agent and applied a controllable shear force via a rotating disk to separate a HREE ternary system (Dy, Yb, Y). At pH 6.0, rejection of each ion exceeded 90%. Based on the differences in the critical shear rates of the complexes, they sequentially dissociated, obtaining β(Y/Dy), β(Y/Yb) and β(Dy/Yb) of 12.38, 13.37 and 10.92, respectively.

In summary, from the efficient separation of ions with distinct valences to the electrostatically regulated sieving of LREEs and HREEs, and further to the precise dissociation of adjacent REEs based on shear stability of their complexes, the NF-based separation strategies exhibit a clear evolutionary path commensurate with increasing separation difficulty. Direct NF performs efficiently in systems with substantial valence differences. The complexation electrostatic strategy provides a feasible solution for LREE/HREE separation, and SID technology shows considerable promise for the most challenging adjacent REE separation. Together, these three approaches constitute a multi-level membrane separation toolbox for multi-component REE systems.

### 4.3. NF Recovery of REEs from Real Leachates and Simulated Wastewaters

Despite their distinct origins, real leachates share three common challenges that distinguish them from model solutions: (i) extreme acidity or wide pH variation, from pH ~1 (NdFeB) to pH ~3.5 (red mud) to pH ~4 (fly ash), demanding membrane acid resistance; (ii) high matrix interference, as the concentrations of Fe/Al/Ca/Na are often 2–4 orders of magnitude higher than REEs, necessitating selective rejection of REEs against a massive background; and (iii) severe fouling propensity, as colloidal hydroxides of Fe/Al, residual organics, and fine particles accelerate flux decline. The following case studies illustrate how these challenges are tackled by tailored membrane selection and process integration [110,111].

When treating NdFeB permanent magnet waste, the acidic leachate is typically extremely acidic (pH ≈ 1.0) and carries a high pollution load, containing, besides REE ions, large amounts of iron as well as minor amounts of boron, cobalt, and other coexisting components [112]. Under such strongly acidic and high-fouling conditions, conventional NF membranes are highly susceptible to acid-catalyzed degradation or severe fouling, leading to a sharp decline in separation performance. To address this challenge, the PIP-TDI membrane prepared by Sun [86], with its unique urea carbonyl structure, can effectively inhibit acid-catalyzed degradation, maintaining structural integrity and separation performance even in a harsh environment of pH ~1.0. Compared with the commercial membrane MPS 34, this membrane also exhibits better hydrophilicity and fouling resistance, offering a promising membrane material for the efficient and stable recovery of rare earth metals from NdFeB wastewater.

For red mud leachate, which contains extremely high levels of iron and aluminum matrices and is strongly acidic, an aqueous environment typically transitions from strongly to weakly acidic. The REE concentrations are low, whereas the concentrations of major components such as Fe^3+^, Al^3+^, and Ca^2+^ are far higher than those of REEs, often accompanied by impurities like titanium, sodium, and silicon, as well as trace radioactive elements [113]. Siddiqui et al. [69], targeting red mud from a Turkish iron mine, constructed a combined process involving microwave acid leaching, NF, and supported liquid membrane separation. In the NF step, approximately 90% REE recovery efficiency was achieved under low acidity (pH 3.5) and 12 bar, and maintaining low acidity helped reduce the osmotic pressure and alleviate membrane fouling. The subsequent supported liquid membrane using D_2_EHPA as a carrier performed selective separation. Within 3 h of reaction time, Y exhibited the highest mass flux, and the response surface model identified optimized operating conditions that balanced high recovery with economic viability.

Coal fly ash nitric acid leachate faces the challenge of extremely low REE concentrations, with the concentrations of major elements such as Ca^2+^, Al^3+^, Fe^3+^, and Na^+^ being 2–4 orders of magnitude higher [114]. This solution also contains Mg^2+^, K^+^, and minor impurities like silicon and titanium, representing a typical system of trace disperse REEs with high matrix interference. Kose Mutlu et al. [82] developed a pretreatment and concentration process involving pH adjustment, MF, and NF. In the pretreatment stage, adjusting the pH to 4.0 combined with 0.45 μm MF removed approximately 98% of Fe and 50% of Al. Under optimal NF conditions (pH 3.5, 12 bar), the DK membrane achieved over 92% rejection of REEs while rejecting less than 10% of Na^+^, successfully realizing the selective separation of monovalent cations, and the economically optimal operating point was determined using a cost model. In addition to coal fly ash, phosphogypsum leachate, spent catalyst leachate, smelting slag leachate and leachate from some low-grade REE ores present similar aqueous environments, all characterized by extremely low REE concentrations, extremely high background concentrations of alkali and alkaline earth metals, and a broad acidity range. These systems share the common challenge of efficiently concentrating and purifying REEs from streams with high matrix interference. Considerable yet untapped REE reserves often exist in such secondary resources.

To understand at a more fundamental level the influence of coexisting impurities in acidic sulfate media on NF separation performance and membrane stability, Lopez et al. [70] evaluated the performance of the semi-aromatic polyamide NF membrane NF270 using sulfate-simulated solutions (pH 1.5–3.0) containing Ca^2+^, Al^3+^, and Zn^2+^. The membrane achieved high rejection of metal ions while allowing sulfuric acid to permeate. The rejection data were fitted to a solution-diffusion model, and permeability parameters of individual ions were quantitatively obtained. Furthermore, the NF270 membrane was subjected to accelerated aging by immersion in 1 mol/L sulfuric acid for four weeks. By comparing the separation performance before and after aging, and characterizing the membranes with ATR-FTIR, AFM, and XPS, it was found that although a long-term acid attack caused certain physicochemical changes, the aged membrane still largely retained its separation capability, providing important support for the practical application of polyamide NF membranes in acidic industrial wastewaters containing REEs. The above studies, starting from diverse real water environments and simulated systems, systematically validate the efficiency, selectivity, and stability of NF in REE recovery, and reveal key strategies for enhancing separation by regulating coexisting impurities and acidity, collectively driving the field toward a green, economical, and sustainable direction.

### 4.4. Comparison of NF with Other Separation Processes

Taken together, NF exhibits significant advantages across multiple performance dimensions. Compared with solvent extraction, it operates entirely in the aqueous phase without phase transfer, substantially reducing organic solvent usage and saline wastewater discharge, and inherently aligns with green chemistry principles. Compared with ion exchange, NF is a continuous cross-flow operation with high throughput, free from the capacity bottlenecks associated with resin regeneration and long batch cycles. Compared with electrodialysis, NF requires only pressure as the driving force, consumes less energy, has a higher tolerance for suspended solids and salinity in the feed, and is less prone to abrupt performance drops caused by scaling on the membrane surface. Compared with membrane distillation, NF operates at a low temperature, requires no heat source, and has more controllable equipment investment and operating costs, while also achieving a degree of selective separation during concentration. NF uniquely integrates the ideal features of aqueous phase operation, continuous high throughput, low energy consumption, and the absence of phase transfer. Although it currently falls short of multi-stage solvent extraction in terms of selectivity, it has found irreplaceable application niches in front-end processes, such as preconcentration of REE leachate, acid recovery, and preliminary impurity separation. From an economic standpoint, NF offers clear advantages over conventional REE separation processes, including lower energy consumption (0.5–2.0 MPa vs. multi-stage high-pressure operation), reduced chemical consumption (no organic extractants or stripping agents), and minimized capital expenditure due to its continuous single-phase operation and compact modular design. The primary shortcoming of commercial NF in REE separation remains its limited selectivity toward isovalent ions. Coupling novel NF membrane materials with integrated systems has therefore become the key endeavor to break through the screening bottleneck. In practice, NF is often integrated in series with other separation technologies, as a complementary front-end step that reduces the feed volume and matrix complexity, enhancing the overall efficiency of downstream high-selectivity but low-capacity processes [115].

## 5. Challenges and Future Perspectives

### 5.1. Current Limitations and Challenges

Given the extreme chemical similarity of lanthanides, the crystal radii of adjacent REE ions (e.g., Pr^3+^/Nd^3+^, Dy^3+^/Ho^3+^) differ by merely ~0.01 Å–0.02 Å. The pore size distribution of commercial NF membranes typically ranges from 0.5 to 2 nm, far exceeding these minute dimensional differences, meaning size sieving alone is wholly incapable of discriminating between adjacent REEs. Charge exclusion is equally ineffective toward isovalent Ln^3+^ ions. Without complexation, the separation factors for adjacent REEs usually fall below 1.5, a significant gap from the >2 threshold required for viable multi-stage industrial operation. Although adjusting the pH or introducing chelating agents can alter REE speciation (e.g., forming differently charged complexes) and thereby magnify separation differences to some extent, this inevitably increases the chemical consumption and process complexity. Therefore, designing sub-nanometer precision channels or incorporating specific recognition groups from the membrane material level represents the fundamental breakthrough for efficient adjacent REE separation [116].

The diverse contaminants in REE leachates and refining wastewaters further exacerbate the separation difficulty. These foulants fall into three categories: organic substances (e.g., residual extractants, humic substances, surfactants) that readily adsorb onto membrane surfaces, forming dense organic fouling layers; colloidal matter (e.g., silicates, iron/aluminum hydroxides) that blocks membrane pores via bridging effects; and REE precipitates, when the local pH rises or the feed reaches supersaturation, Ln^3+^ can hydrolyze to form Ln(OH)_3_ or bind with sulfate to generate insoluble salts that deposit on the membrane surface. The synergistic action of these foulants markedly intensifies the concentration polarization, causing the membrane flux to drop. Although chemical cleaning can partially restore membrane performance, frequent cleaning not only elevates the operating costs and shortens the membrane lifespan but also generates secondary waste streams. Hence, developing membrane surfaces with antifouling properties (e.g., hydrophilic modification, low-adhesion coatings) and optimizing the cross-flow hydrodynamics are critical pathways to mitigate membrane fouling. However, surface modification strategies, if not carefully designed, may inadvertently introduce new fouling risks. While charged functional groups are often introduced to enhance Donnan exclusion for multivalent cations, they may simultaneously attract oppositely charged natural organic matter, silicate colloids, and anionic surfactant residues from upstream operations, leading to the formation of dense organic–inorganic composite fouling layers. The presence of divalent cations such as Ca^2+^ further aggravates this issue through charge neutralization and bridging effects. Similarly, incorporation of high-surface-area nanomaterials, such as MXene or COFs, can create nucleation sites or act as “bridges” that promote foulant deposition. The resulting synergistic fouling, arising from simultaneous organics, colloidal hydroxides, and REE precipitates, is substantially more severe than the fouling by any single foulant class. Future studies should therefore systematically evaluate fouling behavior using real leachates rather than model solutions, and develop tailored surface engineering strategies that judiciously balance selectivity enhancement with fouling mitigation.

Furthermore, weathered crust elution-deposited REE ores are typically processed using sulfate leaching systems, where the total ionic strength of the leachate can reach 0.5–2 mol/L. Under such conditions, high concentrations of background ions, such as Na^+^, Mg^2+^, and SO_4_^2−^, significantly compress the electrical double layer at the membrane surface, weakening Donnan exclusion and causing the NF membrane to lose its high rejection capability for REE ions. Concurrently, the high-salinity environment drastically elevates the osmotic pressure, reducing the effective driving force and leading to marked flux decline. More notably, the competitive ion permeation effect (e.g., preferential transport of Na^+^ dragging a small fraction of REE ions) further deteriorates separation selectivity [117]. Since the existing commercial membranes are predominantly designed for low-salinity water treatment, there is an urgent need to develop novel membrane materials that tolerate high ionic strength while possessing reinforced electrostatic repulsion or rigid pore architecture.

Moreover, REE separation flowsheets involve a variety of harsh chemical environments, placing extremely high demands on the chemical stability of membrane materials. The mineral acid leaching stage can reach pH 0–1, while stripping and washing steps involve highly corrosive media, such as 6 mol/L HCl or 2 mol/L NaOH, along with solutions containing high concentrations of fluoride or sulfate, creating exceptionally challenging operating conditions. Commercial polyamide TFC membranes are highly susceptible to hydrolysis in strongly acidic or alkaline environments, leading to delamination of the separation layer, pore enlargement, and eventual collapse of rejection. Recent modifications using polyelectrolyte layers have demonstrated marked enhancement in pH stability, offering a promising route for harsh condition applications [118]. The polysulfone or polyethersulfone support layers can also undergo swelling or degradation at extreme pH values. Even when chemically resistant materials such as SPEEK are employed, their cost and membrane fabrication processes still face considerable hurdles. In addition, ancillary materials within membrane modules, such as sealing adhesives and gaskets, must possess equivalent environmental tolerance. Developing membrane materials that combine high separation performance with extreme chemical stability is a fundamental prerequisite for integrating NF technology into existing REE smelting circuits.

Finally, existing studies have largely focused on single REE or binary REE model solutions, creating a considerable gap with real REE leachates. Actual process streams contain, in addition to target REEs, impurity ions such as Al^3+^, Fe^3+^, and Mn^2+^; anions like SO_4_^2−^, F^−^, and Cl^−^; as well as humic acids, microbial metabolites, residual organic extractants, and other components. These species engage in complex interactions, including interfacial reactions (e.g., Fe^3+^ hydrolysis generating colloids that co-precipitate with REEs), synergistic fouling (organic–inorganic composite fouling layers) and competitive permeation. Yet, to date, long-term operational stability data for real leachates are extremely scarce. Quantitative models for foulant identification and flux decline have not been established, and pretreatment process design for complex matrices remains virtually unexplored. Compounding this issue, marked differences in leachate chemistry among various REE ore types (ion-adsorption clays, bastnäsite, monazite, etc.) mean that the absence of universally applicable process strategies frequently causes laboratory achievements to fail during industrial scale-up.

### 5.2. Future Research Directions

**Figure 6:**
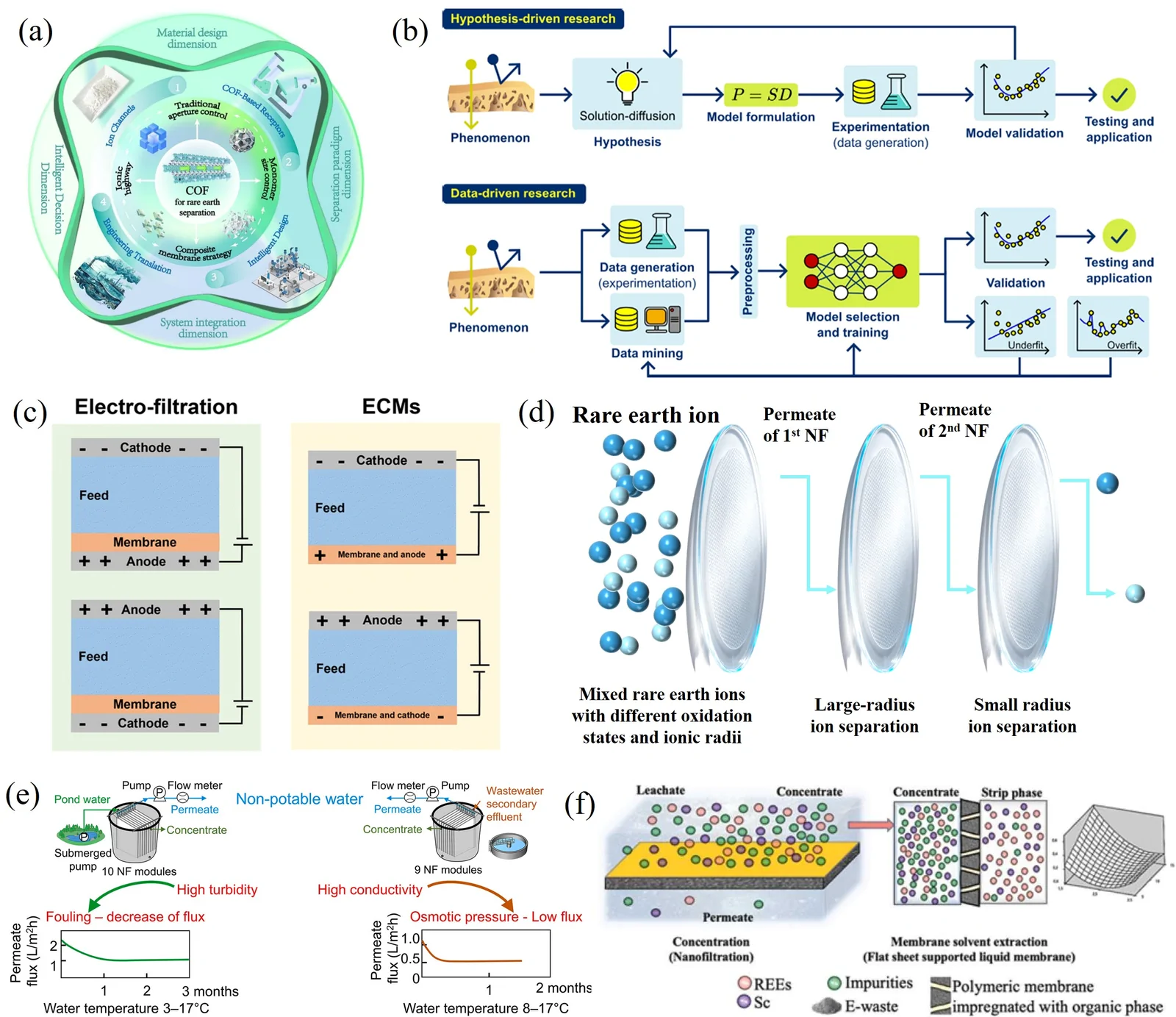
Multi-dimensional design, research paradigms and new membrane separation technologies for rare earth separation from membrane materials: (**a**) A multi-dimensional collaborative design framework for COF-based additive materials for rare earth separation, covering four dimensions: material construction, separation mechanism, system integration and intelligent control [119]. (**b**) Two mainstream membrane separation research paradigms: hypothesis-driven path (modeling and verification based on solution-diffusion model) and data-driven path (experimental data mining, machine learning model training and over-fitting/under-fitting diagnosis) [120]. (**c**) A configuration diagram of electro-filtration and electrode composite membranes (ECMs) is constructed, and the electric field-enhanced separation system is constructed by the differential spatial arrangement of cathode and anode and membrane layers [121]. (**d**) Three-stage series nanofiltration separation of rare earth elements process diagram, according to the progressive difference in ion hydration radius, in turn, to achieve large radius, medium radius and small radius of rare earth ions in the classification of interception. (**e**) Large-scale reconstruction strategy of nanofiltration membrane module and its long-term operation performance evaluation (pilot-scale submerged system) [122]. (**f**) The coupled integrated process of nanofiltration preconcentration and supported liquid membrane solvent extraction shows the complementary advantages of membrane separation and solvent extraction [123].

(1) High-selectivity membrane materials: The key to overcoming the selectivity bottleneck in adjacent REE separation lies in innovative membrane material design. Biomimetic ion channel membranes exploit the sub-nanometer confinement and recognition mechanisms of biological channels, using artificial nanopores constructed from carbon nanotubes (CNTs), metal–organic frameworks (MOFs), covalent organic frameworks (COFs), and other materials to achieve precise ion sieving. The sieving windows of these materials can be precisely tailored to cover the differences in hydrated radii among REEs. COFs feature precisely tunable pore sizes and highly ordered structures (Figure 6a), yet their application as membrane modifiers in REE separation remains largely exploratory, with unresolved issues concerning long-term stability and scalable fabrication [119]. Molecularly imprinted membranes (MIMs), which create target-complementary cavities within the membrane matrix, and surface-grafted functional groups (e.g., phosphate, carboxylate, and picolinic acid moieties) offer additional routes to enhance selectivity via coordination chemistry. The challenge lies not in laboratory scale proof of concept but in scaling up these materials to defect-free membranes with consistent performance over extended operational periods. This hurdle has yet to be surmounted for any COF-based or MOF-based NF membrane. Nonetheless, the prospects are substantial, and continued investment in this direction is expected to yield substantial improvements.
(2) Machine learning and intelligent process control: To cope with feed composition variability and the nonlinear interplay of multiple operating parameters, integrating machine learning and intelligent control offers a transformative pathway for NF process optimization. The current research paradigms are evolving from purely hypothesis-driven approaches (e.g., solution-diffusion model validation) towards hybrid frameworks that incorporate data-driven strategies, including experimental data mining, model training, and overfitting/underfitting diagnostics, into front-end membrane design (Figure 6b) [120]. At the predictive level, models trained on comprehensive datasets (encompassing pH, ionic strength, pressure, membrane type, and REE composition) can accelerate the identification of optimal operating windows. At the real-time control level, online sensors coupled with model predictive control (MPC) algorithms can enable the dynamic regulation of pressure, cross-flow velocity, or pH to sustain a system near its optimum. Such intelligent closed-loop control may not only accommodate feed variations but also mitigate fouling and extend operational lifetimes. Looking forward, the incorporation of deep reinforcement learning could drive NF systems toward increasingly autonomous and self-optimizing behavior. The key barrier remains model robustness and real-time implementation, but the accelerating accumulation of operational data is steadily narrowing this gap.
(3) Coupled intensification technologies: Given the inherent limitations of standalone NF for handling complex REE mixtures, coupling NF with complementary physical or chemical processes presents a promising avenue for synergistic performance enhancement. Electro-nanofiltration (ENF), which superimposes an electric field across a membrane, can harness electrokinetic effects to either facilitate or hinder ion transport while mitigating concentration polarization (Figure 6c) [121]. Another direction is the development of NF adsorption bifunctional membranes, which embed selective adsorbents within a membrane matrix for deep capture of specific REEs, with the potential for in situ regeneration. Beyond these, hybrid configurations such as NF extraction and NF ion exchange are conceptually attractive, though their practical feasibility and long-term stability remain to be systematically validated. Furthermore, multi-stage membrane cascades, which combine NF modules in series or parallel, offer flexible flowsheet designs, intermediate recycling, and scalability, making them particularly suited for multi-component REE separations (Figure 6d). The optimization of cascade complexity and associated capital costs warrants careful consideration, yet the modular nature of NF makes it inherently amenable to such hybrid designs.
(4) Module engineering and process scale-up: Transitioning NF from pilot-scale demonstration to engineering reality requires sustained efforts in module fabrication and process scale-up. Recent pilot studies have shown that reconfigured commercial membrane elements can be adapted for submerged NF operation, demonstrating technical feasibility (Figure 6e) [122]. Nevertheless, several critical issues remain insufficiently addressed. Long-term continuous operation with real REE leachates, extending over hundreds to thousands of hours, is still needed to evaluate flux decline, cleaning intervals, membrane lifespan, and overall energy/chemical consumption. Module configuration also involves inherent trade-offs: series connections favor stepwise purification, whereas parallel arrangements enhance throughput; advanced cascade modes (e.g., diafiltration and concentration) deserve further exploration for progressive enrichment of light and heavy REEs. Beyond geometry, robust membrane housings, acid-resistant seals and effective online cleaning protocols are equally critical for reliable large-scale deployment. Fouling-resistant module design and online cleaning strategies must be integrated from the outset, as they directly determine the long-term operational viability. While NF has already found industrial use in acid recovery, its application to REE leachate preconcentration and impurity removal is still nascent, offering ample room for technological refinement. Furthermore, a systematic techno-economic assessment and life-cycle analysis should accompany membrane development to identify cost-effective fabrication routes and ensure economic viability alongside separation performance. Ultimately, progress in membrane module manufacturing and process scale-up will be decisive for the economic viability and operational resilience of NF-based REE separation systems.
(5) Standardized evaluation framework: A major obstacle to the rapid advancement of NF-based REE separation is the lack of a unified characterization framework, which currently hampers meaningful cross-study comparisons. A standardized evaluation protocol should, at least, include: the separation factor (α) for selectivity; the REE recovery (R%) and product purity (P%) for yield and quality; the flux decline rate (FD%) and recovery rate (FR%) for fouling and cleanability; and the specific energy consumption (kWh/kg REE) for benchmarking against conventional technologies. Baseline conditions (e.g., 25 ± 1 °C; cross-flow velocity, 0.3–0.5 m/s; transmembrane pressure, 1.0 MPa; pH ≈ 3.0) and reference binary systems (e.g., La/Nd and Nd/Dy) are recommended to ensure consistency. Additionally, high-precision analytical tools, notably ICP-MS, are essential for accurate trace-level REE quantification, providing a reliable analytical basis for cross-laboratory comparisons. The primary challenge is achieving broad consensus on these protocols, but the benefits, including direct comparison of results across studies and accelerated identification of truly promising membrane materials, far outweigh the coordination costs.
(6) Closed-loop system integration: Ultimately, NF should be envisioned as a central node in a closed-loop REE smelting system, contributing to the environmental sustainability of the entire chain. A conceptual integrated flowsheet might comprise: leaching, pretreatment, NF preconcentration and grouping, solvent extraction, crystallization, NF recovery of acid and residual REEs from mother liquor, and compliant discharge. Within this architecture, NF could perform multiple functions, including preconcentrating REEs while rejecting impurities (e.g., Al^3+^, Fe^3+^), pre-grouping light REEs (La-Nd) from their medium/heavy counterparts (Sm-Lu) via hydration radius differences, recovering free acids from stripping streams, and treating mother liquor for recycling toward near-zero liquid discharge. This integrated concept has been preliminarily illustrated by hybrid processes combining NF preconcentration with supported liquid membrane extraction (Figure 6f), which exploit the complementary strengths of both technologies [123]. However, the actual industrial viability of such integrated flowsheets requires further validation under realistic feed conditions, and the trade-offs between process complexity and overall efficiency must be systematically quantified. When coupled with intelligent control and heat integration, these systems may offer additional reductions in energy use and environmental footprints. In essence, positioning NF as both a “pre-grouping” and “post-recovery” unit preserves the high selectivity of solvent extraction while reducing organic solvent consumption, acid/base usage, and waste generation at the source, representing a promising direction for sustainable REE manufacturing. The key challenge is that such integration demands simultaneous progress across multiple fronts—membrane materials, process control and module engineering—rather than sequential development, making it one of the most challenging and promising among the six directions.

The six directions outlined above, ranging from membrane materials with high selectivity and intelligent process optimization to coupled intensification technologies, pilot demonstration and standardization, and closed-loop integration across the full process, are interwoven and advance in a layered manner. Among these, the development of chemically robust precision membranes at the sub-nanometer scale remains a critical bottleneck, because without such membranes, even the most sophisticated control algorithms and process integrations cannot overcome the fundamental selectivity ceiling. However, the timeline for such materials innovation is necessarily long. In the interim, process intensification, systematic data generation, and standardized testing will provide the indispensable engineering foundation and empirical feedback that will guide and accelerate the development of next-generation membranes. In the coming decade, with the deep convergence of materials chemistry, membrane science, and artificial intelligence, NF is poised to evolve from a laboratory auxiliary role into a mainstream unit operation, providing robust technological support for the green transformation of the REE industry.

## 6. Conclusions

In summary, NF technology exhibits the core advantages of low energy consumption, absence of phase transition, and environmental friendliness in REE extraction and separation, aligning with green chemistry principles. To address the selectivity challenge arising from the extremely small differences in hydrated radii between adjacent REE ions, CNF has emerged as an effective strategy to overcome this bottleneck by modulating REE speciation and amplifying charge differences. Nevertheless, the maturity of current NF technology remains at a transition stage from pilot-scale to industrial demonstration, and a considerable gap remains. Membrane fouling and deterioration of rejection caused by complex components in real leachates, such as high salinity, strong acids, organics, and colloids, severely constrain its long-term stable operation. Therefore, future research should focus more on real REE feed systems, systematically elucidate the synergistic fouling mechanisms, and develop antifouling membrane materials and online cleaning strategies to accelerate the transition of NF from laboratory to industrial applications and realize the green and efficient separation of REEs.

## Figures and Tables

**Table 2:** Characteristics of the commercial NF membranes.

Membrane Type	Material & Characteristics	MWCO (Da)	Surface Charge	Typical Performance for Rare Earth Separation
NF270 (Dow)	Semi-aromatic polyamide (piperazine based)	200–400	Negative	High metal rejections (>98%) [79]; R_La_^3+^ > 97% [80].
NF90 (Dow)	Dense polyamide membrane	200–400	Negative	High rejection towards polyvalent REE cations; S_Na_^+^_/Ca_^2+^ = 1.59, S_Ca_^2+^_/Ce_^3+^ = 2.03 (pH 6) [81].
NF5 (Dow)	Polyamide membrane	500–800	Negative	Suitable for divalent/multivalent cation separation; S_Na_^+^_/Ca_^2+^ = 1.14, S_Ca_^2+^_/Ce_^3+^ = 6.79 (pH 6) [81].
DK (GE)	Polyamide composite membrane	150–300	Negative	Polyvalent cation rejection > 90%; R_MgSO4_ > 96%, R_REEs_ > 90% (12 bar, pH 6) [82].
DL (GE)	Polyamide membrane	300–500	Negative	Higher flux than DK, suitable for preconcentration; acid recovery 83%, metal rejection 92% [79].
TS80 (TriSep)	Aromatic polyamide membrane	150~400	Negative	Maximum cation rejection at pH < 3; MgSO_4_ 99.0%, NaCl 80–90% [83].
NTR-7450 (Nitto Denko)	Negatively charged sulfonated polyethersulfone (SPES) membrane	~1000	Negative	Salt rejection ~50%; In^3+^ rejection > 98% (neutral pH) [84].

## Data Availability

No new data were created or analyzed in this study. Data sharing is not applicable to this article.

## Nomenclature

Abbreviation	Full Name
REE	Rare earth element
NF	Nanofiltration
LREE	Light rare earth element
HREE	Heavy rare earth element
EV	Electric vehicle
MF	Microfiltration
UF	Ultrafiltration
RO	Reverse osmosis
ED	Electrodialysis
MWCO	Molecular weight cut-off
HF	Hartree–Fock
MP2	Second-order Møller–Plesset perturbation theory
EDTA	Ethylenediaminetetraacetic acid
DTPA	Diethylenetriaminepentaacetic acid
NTA	Nitrilotriacetic acid
CNF	Complexation nanofiltration
IP	Interfacial polymerization
LCA	Life-cycle assessment
ICP-MS	Inductively coupled plasma mass spectrometry
PAH	Poly (allylamine hydrochloride)
MMM	Mixed matrix membrane
O-MoS_2_	Oxidized molybdenum disulfide
PAN	Polyacrylonitrile
PSF	Polysulfone
TMC	Trimesoyl chloride
CNT	Carbon nanotube
SID	Shear-induced dissociation
DFT	Density functional theory
XPS	X-ray photoelectron spectroscopy
AFM	Atomic force microscopy
ATR-FTIR	Attenuated total reflectance FTIR
SPEEK	Sulfonated poly (ether ether ketone)
MOF	Metal–organic framework
COF	Covalent organic framework
MIM	Molecularly imprinted membrane
MPC	Model predictive control
ENF	Electro-nanofiltration
TEA	Techno-economic analysis
